# Antiplasmodial Property of *Glycyrrhiza glabra* Traditionally Used for Malaria in Iran: Promising Activity with High Selectivity Index for Malaria

**Published:** 2018-06-13

**Authors:** Ali Ramazani, Mahdi Tavakolizadeh, Samira Ramazani, Hamidreza Kheiri-Manjili, Mehdi Eskandari

**Affiliations:** 1 Zanjan Pharmaceutical Biotechnology Research Center, Zanjan University of Medical Sciences, Zanjan, Iran; 2 Department of Pharmacognosy, School of Pharmacy, Zanjan University of Medical Sciences, Zanjan, Iran; 3 Department of Physiology and Pharmacology, School of Medicine, Zanjan University of Medical Sciences, Zanjan, Iran

**Keywords:** *Glycyrrhiza glabra*, Malaria, Traditional medicine, *Plasmodium berghei*, Iran

## Abstract

**Background:**

Development of resistance against the frontline anti-malarial drugs has created an alarming situation, which requires intensive drug discovery to develop new, more effective, affordable and accessible anti-malarial agents. The aim of this study was to assess antiplasmodial activity of the different fractions of root extract of *Glycyrrhiza glabra.*

**Methods:**

Roots of *G. glabra* were collected from Tarom district of Zanjan Province in 2016 and then dried root material was chopped and consecutively extracted by the percolation method using solvents of different polarity. Resulting extracts were assessed for in vitro and in vivo anti-malarial and cell cytotoxicity activities.

**Results:**

Among the three different solvent fractions studied, water-methanol and ethyl acetate fractions showed promising in vitro antiplasmodial activity against CQ-sensitive *Plasmodium falciparum* 3D7 strain (IC_50_= 9.95 and 13μg/ml, respectively). Further, the selectivity indices (HeLa cells *versus P. falciparum*) for the promising water-methanol fraction showed selectivity for *P. falciparum* and potential safer therapy for human. Interestingly, water-methanol and ethyl acetate fractions showed a significant suppression of parasite growth (72.2% and 65%, respectively) in comparison with control group in mice infected with *P. berghei* (P< 0.05).

**Conclusion:**

The promising antiplasmodial activity of the aqueous fraction of *G. glabra* obtained in our study warrant bioassay-guided fractionation of this fraction to identify active principles responsible for antiplasmodial activity.

## Introduction

There were approximately 219 million cases of malaria all around the world and 660000 people died from this disease (1). Increasing resistance in the malaria parasite *Plasmodium falciparum* against artemisinin-based drugs is challenging to malaria control programs (2) and demands a wild attempt to develop novel anti-malarial drugs (3–5).

Drugs containing novel structure from natural source represent the main source for the discovery and development of new drugs for malaria (6). The discovery of new anti-malarial drugs from natural sources is increasing after the successfulness of quinine and artemisinin. *Glycyrrhiza glabra* (Liquorice) is part of both western and eastern herbal traditions. It was approved to treat cough, bronchitis, and gastritis by commission E. This plant is used to treat peptic ulcers, asthma, pharyngitis, infections, hepatic disorders and fever (7) and it is one of the main constituents of the anti-malarial ancient Iranian remedy (8).

Previously, based on our research strategies for malaria drug discovery from plant sources (9, 10), we reported the in vivo and in vitro antiplasmodial activity of total root extracts of *G. glabra* (9). Some active compounds with antimicrobial, antiviral and antiprotozoal properties were isolated and characterized from this plant (11–13).

This study was designed to investigate the three solvents fractions of *G. glabra* with different polarities with hexane, ethyl acetate and methanol-water (50:50). In this framework, selected plant was collected and further evaluated for their in vitro and in vivo antiplasmodial activity and toxicity effect on HeLa cells.

## Materials and Methods

### Chemicals

Reagents and materials in this study were obtained from Merck (Darmstadt, Germany) and Sigma-Aldrich (Steinheim, Germany). MTT, FBS and RPMI 1640 medium from Atocel and PAA (Austria).

### Plant material

Roots of *G. glabra* were collected from Tarom district, Zanjan, Iran, at an altitude of 750m. The specimen was authenticated by Dr M Tavakolizadeh and a voucher specimen is deposited at the Herbarium of School of Pharmacy, Zanjan University of Medical Sciences (voucher No. 1073).

### Extraction

Two thousand grams of dried root material was chopped and consecutively extracted by the percolation method (three times for each solvent and 72h for each time) using solvents of different polarities such as hexane, ethyl acetate and methanol-water (50: 50). Extractions were performed at room temperature and solutions were concentrated and dried by rotary evaporation under reduced pressure at 40 °C. The dried samples were stored in a freezer at 4 °C for further use in antiplasmodial and cell toxicity assays.

### In vitro cultivation of *Plasmodium falciparum*

Chloroquine (CQ) sensitive strain (3D7) of *P. falciparum* was used and cultured according to the methods (9, 10, 14, 15). Briefly, *P. falciparum* parasites was cultured on human erythrocytes (blood group O^+^) in RPMI 1640 medium supplemented with 0.5g/100ml AlbuMax I, 25mM HEPES, 19mM sodium carbonate, and 30μg/ml gentamicin sulfate, at pH 7.2 in a gas mixture of 91% N_2_, 6% CO_2_ and 3% O_2_. The medium was changed each day.

### In vitro antiplasmodial assays

The extracts of experimental plants were evaluated for their antiplasmodial activity against 3D7 strain of *P. falciparum*. For drug screening, SYBR green I-based fluorescence assay was used (16). Briefly, sorbitol synchronized parasites (100μl) were incubated under normal culture conditions at 2% haematocrit and 1% parasitaemia in the presence or absence of different plant extracts (100, 50, 25, and 12.5μg/ml). CQ was used as positive controls, while 0.4% (v/v) DMSO was used as the negative control. After 48h of incubation, 100μl of SYBR Green I lysis buffer was added to each well and after mixing incubated in the dark at 37 °C for 1h. Fluorescence was measured using an ELISA plate reader (Infinite M200, Tecan) with excitation and emission wavelength bands cantered at 485 and 530nm, respectively. The background reading for an empty well was subtracted to yield fluorescence counts for analysis. The counts were plotted against the logarithm of the drug concentration and curve fitting by nonlinear regression to yield the drug concentration that produced 50% of the observed decline from the maximum counts in the drug-free control wells (IC_50_). The results were validated microscopically by examination of Giemsa stained smears of extract treated parasite cultures.

### Cytotoxic assay on HeLa cells using MTT assay

The cytotoxic effects of active plant extract fractions (ethyl acetate and water-methanol) on HeLa cells were assessed by using MTT (3–2, 5 diphenyl tetrazolium bromide) assay (17–20). The HeLa cells cultured in RPMI medium containing 10% foetal bovine serum and incubated at 37 °C with 5% CO_2_ and 96% humidity. Briefly, cells (10^4^ cells in 100μl of culture medium) were distributed in 96-well flat-bottom plates in complete medium. Drug solutions were added after 24h of seeding and incubated for 48h. After 48h, 20μl of a stock solution of MTT (4mg/ml in phosphate buffered saline) was added to each well, gently mixed and incubated for another 3h. Then supernatant was removed and 100μl of DMSO (stop agent) was added. The absorbance of each well measured at 540nm using an ELISA plate reader (Infinite M200, Tecan). The 50% cytotoxic concentration (TC_50_) of test samples was determined by analysis of dose-response curves. Therapeutic index was calculated as a ratio of TC_50_ HeLa /IC_50_ 3D7.

### In vivo antimalarial assay

In vivo antimalarial activity of different solvent fractions of *G. glabra* assessed using the 4day suppressive test against *P. berghei* infection in mice (21). Female Swiss albino mice, weight 18–20g were inoculated with *P. berghei* (ANKA strain). Each mouse received 10^6^ infected erythrocytes by intra-peritoneal (IP) injection on the first day of the experiment. Groups of five mice were dosed daily by IP injection (200mg/kg) for 4 consecutive days. On day 5 of the test, a blood smear was taken from the mice. Percentage suppression of parasitaemia for the fractions was calculated as 100– [(mean parasitaemia treated/mean parasitaemia control) ×100. For comparison of average parasitaemia, one-way ANOVA and two-tailed Student’s t-test were used (SPSS 21.0 Inc., USA) with P< 0.05 being considered significant. CQ at 25mg/kg was used as a positive control. The solvent (20% DMSO in PBS solution) was used as negative control.

The study was approved by the Institution Animal Ethical Committee.

## Results

### In vitro anti-plasmodial assay and cytotoxic study

All the three fractions of *G. glabra* root extracts were screened for in vitro anti-plasmodial activity against the CQ-sensitive (3D7) *P. falciparum* strain (Table 1). The water-methanol and ethyl acetate fractions showed promising antiplasmodial activity with IC_50_ values of 9.95 and 13μg/ml, respectively. The n-hexane fraction did not show admissible activity. The two above mentioned active fractions (water-methanol and ethyl acetate) further analyzed for their toxicity on HeLa cells. The selectivity index (SI) is defined as the ratio of the HeLa cells toxicity to the antiplasmodial activity and is determined by dividing the TC_50_ values for the HeLa cells by the IC_50_ value for *P. falciparum*. The water-methanol fractions showed low toxicity against HeLa cells with higher selectivity against malaria (Table 1).

**Table 1.** Antiplasmodial activity, cytotoxicity and selectivity of water-methanol and ethyl acetate extracts of *Glycyrrhiza glabra*

**n**	**Extract fraction**	***P. falciparum* IC_50_ (μg/ml)**	**HeLa cell IC_50_ (μg/ml)**	**Selectivity indices**
**1**	Water-methanol	9.95	> 100	> 10
**2**	Ethyl acetate	13	21	1.61
**3**	n-hexane	215	-	-

(−) not tested

### In vivo anti-plasmodial assay

The fractions that showed promising antiplasmodial activity further analyzed for their in vivo anti-plasmodial property (Table 2). The water-methanol and ethyl acetate fractions showed a significant suppression of parasitaemia (P< 0.05). In comparison to control group, the water-methanol and ethyl acetate inhibited 72.2% and 65% of the growth of the parasite, respectively.

**Table 2.** In vivo activities of plant extracts against *Plasmodium berghei*

**Group**	**Dose (mg/kg)**	Mean Parasitemia (SD*)	**% Suppression of parasitemia**	**P-value**
**Negative control (20% DMSO in PBS solution)**		11.55 (2.58)		
**Water-methanol**	200	3.15 (1.08)	72.2	0.03
**Ethyl acetate**	200	4.04 (2.36)	65	0.02
**CQ**	25	0	100	

SD: Standard deviation

## Discussion

Medicinal plants have a wide diversity of medicinal properties including for malaria therapy as two of the most important anti-malarial agents, namely quinine and artemisinin, with plant origin. Traditional plants could be attractive for drug discovery as they are widespread and also a large population.

In the present study, different fractions of *G. glabra* root extract known for its traditional medicinal usage (8) and anti-malarial activity (9, 22) was further evaluated for its different solvent fractions antiplasmodial activity against CQ-sensitive *P. falciparum* 3D7 and *P. berghei* ANKA strain and their toxicity against HeLa cell line (Table 1). Among the three different solvent fractions studied, water-methanol and ethyl acetate fractions showed promising in vitro antiplasmodial activity against CQ-sensitive 3D7 strain (IC_50_= 9.95 and 1μg/ml, respectively). Further, the selectivity indices (HeLa cells *versus P. falciparum*) for the promising water-methanol fraction showed selectivity for *P. falciparum* and potential safer therapy for human. Interestingly, water-methanol and ethyl acetate fractions showed a significant suppression of parasitemia in comparison with control group (P< 0.05). The suppression rate of parasite growth induced by water-methanol and ethyl acetate fractions were 72.2% and 65%, respectively (Table 2).

In our previous study (9), the hydro-alcoholic extraction of *G. glabra* root extract showed promising antiplasmodial activity with IC_50_ values of 13.56μg/ml against *P. falciparum* 3D7 strain and also suppressed the growth of the *P. berghei* parasite by 65% in vivo at a dose of 400mg/kg. In this study, further fractionation resulted in better antiplasmodial property in vitro and in vivo. Water-methanol fraction exhibited in vitro antiplasmodial activity with IC_50_ value of 9.9μg/ml and suppressed the growth of the *P. berghei* parasite by 72.2 % at a dose of 200mg/kg in comparison with the control group. The active ingredients of plant accumulated in water-methanol fraction. The ethyl acetate fraction also showed promising in vitro and in vivo antiplasmodial activity, but this fraction had low selectivity index in comparison with water-methanol fraction. Licorice (*G. glabra*) is used for the treatment of several diseases in traditional medicine. Licorice was shown to have a number of biological properties such as antiviral, anti-inflammatory, antimicrobial, and anticancer activities (13). Several active components from *G. glabra* were isolated and some cellular and molecular mechanisms of these compounds have been elucidated (11, 12, 22). One of these compounds is Licochalcone A, separated from the roots of Chinese licorice, showed promising in vitro antiplasmodial property against both CQ-susceptible (3D7) and CQ-resistant (Dd2) *P. falciparum* strains. This compound protected the mice from the lethal *P. yoelii* infection (22). 18beta-glycyrrhetinic acid, another anti-malarial agent isolated from Indian licorice, showed significant in silico, in vitro and in vivo antimalarial activity (12).

## Conclusion

The water-methanol fraction of *G. glabra* has higher selectivity index for malaria than ethyl acetate fraction. The promising results with the aqueous fraction of this plant obtained in our preliminary study, warrant bioassay-guided fractionation of this fraction to identify active principles responsible for antiplasmodial activity.
